# Study protocol of an effect and process evaluation of the Stamina model; a Structured and Time-effective Approach through Methods for an Inclusive and Active working life

**DOI:** 10.1186/s12889-018-5807-9

**Published:** 2018-08-29

**Authors:** Magnus Svartengren, Therese Hellman

**Affiliations:** Department of Medical Sciences, Occupational and Environmental Medicine, Uppsala University, Dag Hammarskjölds väg 60, 752 37 Uppsala, Sweden

**Keywords:** Work environment management, Organization, Qualitative method

## Abstract

**Background:**

The working environment should be a naturally integrated part of business development. Provisions are in place that address the employer’s obligations to investigate, carry out and follow up activities in such a way that ill health and accidents at work are prevented and a satisfactory working environment is achieved. Still, there are organizations that not properly perform systematic work environment management. In order to improve adherence, interventions and models focused on these issues need to be easy to understand and provide rapid feedback of results in order to be implemented. The Stamina model has recently been implemented in Sweden. The model is a participatory organizational systematic model facilitating the work environment, productivity and quality. It is a support model that provides structured and recurrent feedback in the systematic work environment management. The aim of the present paper is to thoroughly describe the Stamina model and the studies that are designed to investigate the effect, to increase the understanding of how and why the model was or was not effective, and to identify factors that facilitate implementation.

**Methods:**

The paper presents a project consisting of two related evaluation parts. Part one is an effect evaluation with an active group applying the Stamina model and a control group. This part investigate effects on proxy outcomes that are relevant for health and productivity. Part two is a process evaluation with a qualitative design. This part will be based on semi-structured interviews with various stakeholders, such as employees, first line managers, project managers, facilitators and representatives from the management group, in the organizations.

**Discussion:**

Many interventions found to be effective in research projects fail to translate into meaningful outcomes across multiple contexts. In this project a participatory approach will be adopted, including the possibility to modify the model according to organizational needs and preconditions. Valuable knowledge regarding the design and implementation of the model will be generated in order to develop a model that is suitable and sustainable in organizations.

**Electronic supplementary material:**

The online version of this article (10.1186/s12889-018-5807-9) contains supplementary material, which is available to authorized users.

## Background

Work-related illnesses and accidents are issues that cannot be neglected in society because they have great consequences for both the individual worker [1] and the employer [2]. On a global level, it is estimated that 317 million people suffer from work-related illness and accidents annually [2]. In the late 1980s, employers’ responsibility was clarified in a European directive stating that employers are required to assess risks to the safety and health of workers, and to implement measures aimed at reducing the identified risks [3]. The working environment should be a naturally integrated part of organizational development. National legislation regulating employees’ health and safety at work varies across countries around the world. In Sweden, there is the provision of Systematic Work Environment Management (AFS 2001:1) [4], which addresses employers’ obligations to investigate, carry out and follow up activities in such a way that ill health and accidents at work are prevented and a satisfactory working environment is achieved. Although this guideline is available, it has been reported that there are still organizations in Sweden that do not properly perform their systematic work environment management [5]. These findings might be interpreted in light of other research showing that interventions focused on these issues need to be easy to understand and adopt in the organization if they are to be implemented [6], and it is known that the Systematic Work Environment Management provision is often experienced as time-consuming and somewhat abstract [7–9]. Furthermore, it is also known that health preventive workplace interventions that lack rapid feedback on results causes organizations to be less interested in focusing on work environment management [6]. Another key to successful implementation of interventions focused on systematic work environment management is the engagement of first-line managers, because they are in the position and have opportunities to influence the work environment by providing support to employees [10]. It is well-known that the support given by first-line managers positively influences employees’ job satisfaction, work engagement [11] and perceived work environment [12]. However, central initiatives taken in organizations do not always reach first-line managers and their work groups.

Nielsen and colleagues [13] compiled an overview of five European methods/interventions aimed at improving employee health and wellbeing using systematic approaches. They identified five phases with certain elements that are of importance for an effective intervention. These phases are: preparation, screening, action planning, implementation and evaluation. Organizational readiness for change [14] and effect evaluation are examples of important elements [13]. Furthermore, it has been found that employee participation was an overarching theme that was apparent in all phases and elements [13, 15]. It has been emphasized that, to promote employee participation, employees’ involvement needs to be in place already in the preparation phase [13]. Employee participation have also been highlighted in terms of being involved in problem-solving and decision-making to promote mental health at work [16].

The Stamina model has recently been implemented in Sweden in order to concretize systematic work environment management. Stamina is an acronym for “Structured and Time-effective Approach through Methods for an INclusive and Active working life”. The model that builds upon the Model of Integrated Group Development [17, 18] has a participatory systematic approach aimed at improving the work environment, productivity and quality. It is a support model that provides structured and recurrent feedback in the systematic work environment management. Evaluating a model focused on the organizational level – and involving employee participation as a central part and direct engagement from organization stakeholders – entails some challenges; for example, the researcher has less control over the action that is taken. Still, the Stamina model deliberately takes this stance, which has been supported in previous research showing that work environment management interventions often are complex organizational interventions that need to be adapted to the specific organization. Even though such adaptations are made, it is possible to conduct an effect evaluation using a controlled design [19]. It might also be appropriate to broaden the focus from solely providing knowledge on effectiveness to generating knowledge that could guide forthcoming actions based on a certain model/intervention [20] as well as to increase the understanding of why and how the model/intervention is or is not perceived as effective [21]. Such an approach will provide opportunities for organizations to continue to adapt the efforts and actions to their real-world context. To generate useful knowledge about such flexible models from research, it is important to thoroughly describe the framework of the actual model, and the context for the study.

The aim of the present paper is to thoroughly describe the Stamina model and the studies that are designed to investigate the effectiveness of the Stamina model, as well as why and how this model is or is not experienced as effective.

## Methods/design

The paper presents a project consisting of two related evaluation parts. The aim of the first part, the effect evaluation, is to investigate effects on proxy outcomes that are relevant for health and productivity. The aim of the second part, the process evaluation, is to increase the understanding of how and why the intervention was or was not effective, and to identify factors that promote implementation of the Stamina model.

The study is approved by the Regional Ethics Committee in Uppsala, Sweden (Reg. No. 2017/093).

### The Stamina model

The description of the Stamina model is inspired by the template for intervention description and replication (TIDieR checklist) [22].

#### Materials and procedures used in the Stamina model

The basic principle of systematic work environment management and organizational development is that it should be a perpetual, repetitive process for improvement. The idea behind the development of the Stamina model is that it should facilitate employee participation and rapid and recurrent feedback to the first-line managers and their work groups. The work process in the Stamina model is owned by the organization and is not dependent on any external consultants or researchers. In practice, this means that organizations can arrange their work in various ways, leading to some modifications of the model. One example is that the sessions within the model might be delivered either by a facilitator or a first-line manager, or by these two actors together. This approach makes the actions taken by the organizations less controlled than is typical in this kind of research. Some elements are still fixed and these will be presented below.

The Stamina model sessions are delivered three times annually during the project’s two-year duration. The first session (workshop) lasts for approximately 3 h and the second and third sessions (follow-ups) for 1 h each. The sessions are held at four-month intervals. All three sessions are preceded by a web-based questionnaire measuring Human Resource Index (HRI). The basic question reads “What characterizes your current work situation” and includes an evaluation scale indicating whether the factors are positive or negative and a scale indicating whether the respondent can influence the situation. All employees answer the question with free text and, thus, have the opportunity to emphasize aspects they feel are the most important at that moment. Reports are generated, based on these questionnaires, and serve as working material to support the work group’s reflections and discussions during the sessions.

The first session (workshop) consists of: 1) reflections on the shared basic values, aims and goals of the work group, 2) reflections on the work group’s current work situation, 3) reflections on how the work group wants their work situation to be, and 4) reflections on what actions can be taken to create the desired work situation. In the last step, the work group prioritize one activity they want to focus on and create an action plan based on a manual. The second and third sessions (follow-ups) include a review of previous action plans and creation of new ones.

#### Description of the expertise and specific training given to the providers

In the Stamina model, the workshops are delivered by facilitators working in the participating organizations. It is the organizations that decide which persons that is eligible for the role of facilitator. These persons might, for example, work at a central Human Resource unit within the organization or closer to the specific work groups. Having the opportunity to choose who will be the facilitators increases the feasibility of and adherence to the model in the organization. The two follow-ups are led by the facilitator or the first-line manager in the organization, with support from the facilitator.

The facilitator attend a two-day course to learn how to lead the workshop and how to support the participants in creating the action plans. Between the two course days, the facilitators conduct a pilot workshop in which they practice their role as moderator/facilitator of the session. On the second day, their experiences from the workshop are discussed and reflected upon. The first-line managers do not receive any specific training other than videos with instructions on how to run the sessions. They always have the opportunity to ask and discuss specific issues with the facilitators.

#### Mode of delivery

The sessions in the Stamina model are provided to a group of employees, all of whom work together. The size of the groups depends on the number of employees included in the work groups, which may vary across the organization types targeted. Approximately 8–20 employees is an acceptable size for a group.

The sessions are held at the participants’ own workplaces. The first-line managers choose a suitable room for the sessions in collaboration with the facilitator.

#### Tailoring of the Stamina model

The sessions take a generic approach during the first year to enable the participating organizations to learn the structure and overarching content of the sessions. This is the same for all participants/work groups. However, tailoring is built into the intervention, as a variety of activities are identified and in focus during the work with action plans. Furthermore, the Stamina model during year two can be tailored so as to be feasible in practice for the organizations and work groups. This tailoring may involve, for example, a change in the person leading the workshop (from facilitator to first-line manager) or in session length. Such tailoring is built into the model to facilitate long-term sustainability and implementation adapted to different organizations. However, the content of each session from year one should be retained.

### Part 1 - effect evaluation

#### Study design

This is a quantitative study with a non-randomized waiting-list pre-post trial design. Work groups are assigned to either a control group that only answers to the web-based questionnaire that is the first action within the model or to an active group that conduct all parts included in the Stamina model. The control group that only answer the web-based questions will be given the opportunity to conduct all parts of the model at a later point.

#### Aim and research questions

The aim of this evaluation is to investigate effects on proxy outcomes that are relevant for health and productivity in the active and control group. These proxies are HRI, perceived productivity, organizational justice and sleep.Research questions include the following:

1. How are HRI, perceived productivity, organizational justice and sleep changing over time in the active and control group?
2. What characterizes groups with high and low HRI at the start of the intervention?
  - What types of focus areas are identified in the groups?
  - Does HRI correlate with sleep and organizational justice in the work groups?

#### Study sample

Municipalities in Sweden are eligible for the study. Recruitment of municipalities that are eligible to participate in the project will be a country-wide process. The eligible municipalities will be selected so as to achieve variation in number of employees and geographical conditions. Approximately 20 municipalities will be included in the STAMINA project.

Before the municipalities make their final decision regarding participation, they will be involved in a pre-programme focused on establishing commitment, preparation and planning [13]. The programme includes a lecture on work environment, establishing commitment in the respective organizations and discussions with the research group.

Based on the perspective that the project has its point of departure in the real-world context, the participating municipalities have the opportunity to choose the number of employees included in the project. The number of employees might vary between 100 and 1000. Employees representing approximately 20% of the total number of employees using the model in each municipality will be allocated to the control group. This control group size will be sufficient to detect general trends in the study population. A power calculation of the study groups with the given sample sizes, estimated standard deviation of 29 and a power of 0.8 will detect a difference of mean of HR index at 0.9 units.

The study sample will consist of work groups applying the model in their systematic work environment management. That is, no information regarding the individual employees will be analysed. Demographics will be presented regarding the work groups characteristics. Considering the preliminary numbers of municipalities and employees included in the project, the study sample is estimated to include between 500 and 1800 work groups in the group working according to the Stamina model.

#### Recruitment procedure

The recruitment process is taken place in several steps. The first step includes oral and written information given by the research group to representatives from the management groups in the municipalities. Informed consent forms signed by a person from the management group are delivered to the principal investigator of the project.

The recruitment of first-line managers and their work groups is an internal process in each municipality. The persons representing the management groups perform this recruitment process in ways suitable and adapted to their own organizations. It is during this process work groups are assigned to either the active or control group and this is done based on individual criteria in each municipality.

#### Test instruments

The questionnaire contains questions on Human Resource Index, perceived productivity, organizational justice and sleep. These questions are described below.

##### Human resource index

The Human Resource Index (HRI) measures employees’ perceptions of their current work situation. HRI expresses a value on a scale ranging from 0 to 100 and is calculated based on a free text question, in which employees are asked to identify what characterizes their current work situation. The value represents the individuals’ perception of their work situation based on two parameters: 1) if the experience is positive or negative and 2) perception of opportunity to influence the situation. This index is one of several indices established and shown to predict risk of negative health outcomes [23].

##### Perceived productivity

Perceived productivity is measured using two validated questions that capture the effect of health problems and work-related problems on work performance [24–26]. The questions are formulated: “During the past seven days, how much did your health problems/work environment problems affect your performance while working?” The employees are asked to rate their work performance on a scale ranging from 0 to 10, where 0 = “Health problems/work environment problems had no effect on my work” and 10 = “Health problems/work environment problems completely prevented me from working”.

##### Organizational justice

Organizational justice has previously been used to indicate work environment characterised by sustainable employee health. Organizational justice is divided into two dimensions: procedural and relational justice [7, 8, 27]. In the present study, the focus is on relational justice, which refers to how employees are treated by their superiors, e.g., whether employees’ personal viewpoints and rights are considered and whether employees are treated in an unbiased manner, truthfully and with kindness. There are five statements concerning relational justice; the participants are to respond by indicating to what degree they agree or disagree with the statements. The scale ranges from 1 to 5, where 1 = strongly disagree and 5 = strongly agree.

##### Sleep

Sleep is assessed using a single item from the Karolinska Sleep Questionnaire [28]. The instructions to the participants are to rate whether they have experienced the following complaint during the past three months. The statement is formulated: “Do not feel refreshed when waking up” and is assessed on a rating scale with six response alternatives, from never to always. The sleep question is used to estimate short-term recovery.

#### Data collection

The participants in the group that work according to the Stamina model are answering the short questionnaire on six occasions at four-month intervals. That is, the data collection is following the time points built into the model. The first data collection point (baseline) is immediately before the first three-hour session (workshop), and the following data collection points are in conjunction with the recurrent sessions at four, eight, 12, 16 and 20 months from baseline. The control group answers the same questionnaire on three occasions: at baseline, 12 and 20 months (see Additional file 1: Figure S1).

The questionnaire is administrated over the Internet and sent out to the employees through a digitalized system.

#### Analysis

Statistical methods adapted to a non-randomized waiting-list controlled pre-post trial design will be applied. HRI is an estimate of group resilience and a predictor of future health status in work groups and is also used as guidance for group development. Given the emphasis on the work group, all analyses will be performed at the group level, where each work group is treated as one unit/individual. Repeated measures will be analysed using generalized linear models to detect changes over time.

### Part 2 - process evaluation

#### Study design

This is a qualitative study with an exploratory longitudinal design, including data from semi-structured interviews as well as documents.

#### Aim and research questions

The aim of this process evaluation is to increase the understanding of how and why the intervention was or was not effective, and to identify factors that promote implementation.Research questions:

1. How do representatives from the management groups reason when deciding to engage in the Stamina model and what incentives influenced their decision?
2. How was the intervention delivered by the facilitators and how did they experience the use of the intervention?
3. How did first line managers experience the use of the Stamina model and how this work influence the work environment management in their work groups?
4. How did the employees experience the involvement in the Stamina model?
5. Did the intervention change the employees’ engagement and participation in work-environment-related issues at their workplace?
6. What factors promoted implementation and long-term use of the Stamina model?

#### Study sample

To generate knowledge regarding how the Stamina model works and what factors promote implementation, it is important to gather information from several sources to understand the issue in focus from all perspectives represented in the organization [21]. This is taken into consideration in the present project by interviewing persons in various positions. The process evaluation involves a subset of participants from the effect study. One case in each municipality is longitudinally followed during the project period (see Additional file 1: Figure S1). Each case includes a group of employees, their first-line manager, the project manager, a facilitator and a representative from the management group.

#### Recruitment procedure

Participants are recruited in collaboration with the project managers in the municipalities. The recruitment of work groups is based on a purposeful criterion sampling strategy [29]. In this sampling strategy participants that meet certain criteria are approached in order to achieve variation. In this study, the participants that has certain roles (e.g. first line managers, facilitators) and works in various working areas (e.g. elderly care, preschool or technical work) will be asked to participate. The work group of employees constitutes the basis for further inclusion.

Written informed consent is signed by all participants at the time of the first interview. All participants are informed that participation in the study is voluntary and that they can withdraw from the study at any given time, without giving any reason for their decision.

#### Data collection

Longitudinal focus groups (work groups) and individual semi-structured interviews (first-line manager, project manager, facilitator, representative from the management group) will be conducted. Using focus group interviews in the work group is a natural choice, because the focus group method is well suited to homogenous groups of participants who have a shared framework and experiences based on similar preconditions. Furthermore, having a homogenous group is also helpful in creating a rewarding and informative interview atmosphere, which facilitates generation of new knowledge about and insights into the study area [30, 31]. Individual interviews are conducted with the other actors. Applying a longitudinal design [32] with recurrent interviews during the process of implementing the Stamina model will make it possible to identify changes in the way the model is used and thus increase the understanding of the effective mechanisms. For data collection time points see Additional file 1: Figure S1.

The interviews will be semi-structured, with an interview guide focusing on four themes: 1) expectations of the Stamina model, 2) experiences from the actual work based on the Stamina model, 3) facilitators and barriers concerning working with the Stamina model, and 4) lessons learned for the forthcoming work (interview 2 and 3). The interview guides will be modified for each group of participants, but have the same base and content.

The material will consist of approximately 180 interviews that will be analysed and used to address the various research questions.

Documents produced before and during each session will be gathered for the cases included in the study. For each case, this material will consist of six reports on how the work group experience their current work situation and documents (action plans) concerning what issues they will focus on until the next session. Data derived from the web-based questionnaire in the effect evaluation will also be used in this part of the project. Adopting a case study design that employs both qualitative and quantitative methods is recommended to generate knowledge that can increase the understanding and the explanation of causal mechanisms underlying real-world interventions [21].

#### Analysis

Digital recordings from the interviews will be transcribed verbatim and information about participants’ identity removed. A constant comparative approach inspired by Grounded theory will be used to analyse the transcripts [33]. The analysis will be conducted in several steps focused on the various research questions in the project. All material will be read through several times to create an overall understanding of the data and analysed line-by-line, which will involve marking and assigning a code to the content related to the study aim. These codes should be close to the participants’ own wordings. The analysis of the focus group interviews will not be overly detailed in the first coding, as this may result in loss of valuable information from the discussion and interaction. In these cases, it may be advantageous to assign codes to sequences rather than on a line-by-line basis. The line-by-line and sequence-by-sequence analyses will be conducted and stored in a software program, in order to organize and handle the large amount of data collected. Use of a software program will facilitate the forthcoming steps in the analyses, as it will make it possible to merge the interviews that are relevant to the separate research questions. For example, to answer the research question regarding how the intervention was delivered by the facilitators and how they experienced the use of the intervention, interviews with facilitators will be used. Furthermore, to understand factors that promoted implementation, interviews from all actors in the separate cases (employees, the first-line manager, facilitator, project manager and representative from management group) will be used. Depending on the research question in focus, the codes from the separate interviews will be compared to identify similarities and differences. This comparison will provide the basis for the initial category creation, which will then be discussed in the research group to enhance credibility. Researchers in the research group who have not been included in the first steps of the analysis will pose new questions to the material and provide alternative ways of interpreting and understanding the collected data. Once the research group has reached a consensus on the categories, the analysis process will continue to the next step. The categories from each individual interview will be compared to each other. In this phase, the categories will be raised to a more abstract level with more concrete subcategories. The results will be based on the categories and subcategories. In order to further ensure credibility, the categories will be constantly compared to the basic data, this is, to the transcribed interviews. Furthermore, the research group will meet regularly throughout the analysis process to discuss the emerging codes and categories.

The documents will be analysed by categorizing the areas in focus in the reports and what aspects the employees choose to work with during the sessions.

## Discussion

The current paper presents a participatory organizational systematic model focused on work environment, and the design of the effect and process evaluation. The model is a support model that provides structured and recurrent feedback to organizations that work according to the provisions of the Swedish Work Environment Authority regarding Systematic Work Environment Management (AFS 2001:1) [4] and the Organizational and Social Work Environment (AFS 2015:4) [34]. The provision of Systematic Work Environment Management addresses employers’ obligations to investigate, carry out and follow up activities in such a way that ill health and accidents at work are prevented and a satisfactory working environment is achieved. Although this provision, including guidelines, is available, it has been reported that there are still organizations in Sweden that do not properly perform their systematic work environment management and the guideline is sometimes experienced as abstract. Furthermore, it is known that the guidelines are effective when being used. This project is thus less about the effectiveness and more about how the structured support model might facilitate the use of the Systematic Work Environment Management that already are in place. The Stamina model is designed to provide a structure for this work and to facilitate the engagement of first-line managers. Its key elements are employee participation and rapid and regular feedback to first-line managers and their work groups. With the participatory approach in this research project including the possibility to modify the model according to organizational needs and preconditions, valuable knowledge regarding the design and implementation of the model will be generated.

Many interventions found to be effective in research projects fail to translate into meaningful outcomes across multiple contexts. To increase the knowledge and understanding of the potential poor effect in real-world settings, it is recommended that researchers provide a formative evaluation. Such an evaluation will assess to what extent the implementation is effective in a certain context [35, 36]. It might thus be concluded that performing a process evaluation (formative) is one step forward in an attempt to decrease the gap between research and practice, even though the intervention under evaluation is still often developed from the single perspective of the researcher [36]. We argue that it is important to already in the research project focusing on developing interventions/models that fit into the contexts and preconditions that are in place in organizations in our society. In the present project, we have adopted a participatory approach in order to develop a model that is suitable and sustainable in organizations. This stance implies the allowance of modifications of the model during the second year concerning how the sessions in the Stamina model is delivered, the intention being to match each organization’s unique preconditions. Such an approach is highly relevant in research evaluating work environment interventions [20]. Although the approach is recommended, it also entails some challenges for the research project and the effect evaluation in terms of design, as control over the actual intervention will decrease. However, we argue two things in this regard: 1) that the Stamina model is a systematic organizational approach focusing on systematic work environment, in which it is difficult to precisely define what the active ingredients actually are and how they relate to each other [37] and, for this reason, 2) that the real-world contexts need to be taken into consideration. Hawe, Shiell and Riley [19] argued that interventions that are modified to each participant can nonetheless be evaluated using controlled trials. Furthermore, they suggested that the essential functions of the intervention need to be fixed, while the form of the intervention may be allowed to vary in different contexts. This reasoning is in line with how the Stamina model will be delivered and performed in the participating organizations.

One challenge in the effect evaluation is the comparison between the active and the control groups included in the study. This issue is evident because the HRI is used both as a tool within the Stamina model and as a test instrument measuring employees’ perceptions of their current work situation [23]. The difference between the groups is that one group continues to work with the answers from the HRI in a structured way according to the Stamina model and the other group do not have any structured follow up. Still, it is known that only measuring might highlight and put the issue on the agenda. However, the project also generate longitudinal data that enable comparisons over time in the group using the Stamina model.

For the process evaluation it is important to involve several organizational actors and their mental models in order to fully understand intervention outcomes [21], and employing this approach may be seen as one strength of the present project. Several types of qualitative research have been considered, such as grounded theory, phenomenology, narrative and an ethnographic approach for this part of the project [38]. The application of grounded theory is suitable in areas where there is a lack of knowledge and in areas that have not been previously explored [39]. Furthermore, it is a suitable method to apply when studying processes [33, 39], and that is the focus of this part of the project. Grounded theory is also suitable when multiple data sources are used (in this case interviews, documents and questionnaires). Furthermore, the use of memo writing is of value in the project given the large amount of data that will be collected. However, grounded theory also includes the notion that theory should be generated and that is not the goal of the present project. In summary, we find the analysis procedure of the constant comparative approach to be well suited to the project, though it is not a full-scale grounded theory study. Hence, the material will be analysed using a constant comparative approach, which is often used in grounded theory studies [33].

## Additional file

Additional file 1: Flowchart of the Stamina project. (JPEG 65 kb)

## Abbreviations

HRI: Human Resource Index
Stamina: Structured and Time-effective Approach through Methods for an INclusive and Active working life
TIDieR: The template for intervention description and replication
