# Supplementary figures and images for: Study protocol of an effect and process evaluation of the Stamina model; a Structured and Time-effective Approach through Methods for an Inclusive and Active working life
Source: BMC Public Health. 2018 Aug 29;18:1070. doi: 10.1186/s12889-018-5807-9 (PMC6114887; doi:10.1186/s12889-018-5807-9)

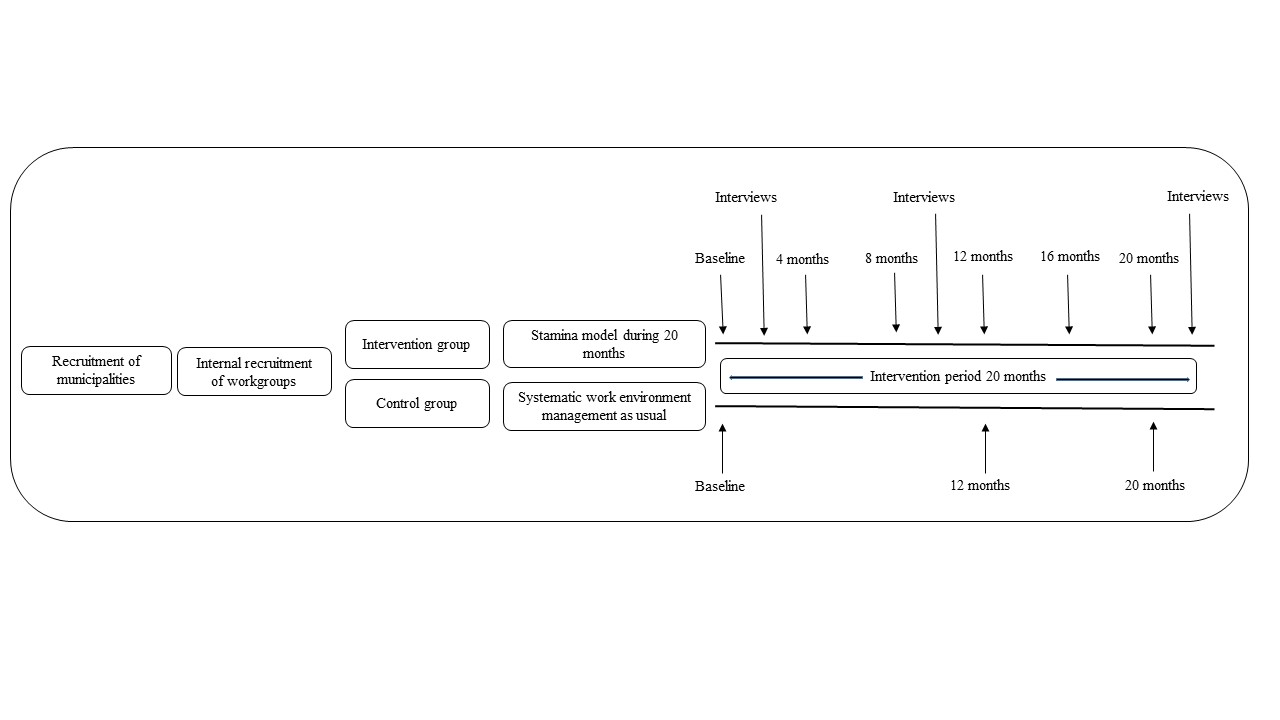

Supplement: Supplementary file 1 — Flowchart of the Stamina project. (JPEG 65 kb) [file 12889_2018_5807_MOESM1_ESM.jpg]
